# Realization of Oriented and Nanoporous Bismuth Chalcogenide Layers via Topochemical Heteroepitaxy for Flexible Gas Sensors

**DOI:** 10.34133/2022/9767651

**Published:** 2022-06-23

**Authors:** Zhiwei Wang, Jie Dai, Jian Wang, Xinzhe Li, Chengjie Pei, Yanlei Liu, Jiaxu Yan, Lin Wang, Shaozhou Li, Hai Li, Xiaoshan Wang, Xiao Huang, Wei Huang

**Affiliations:** ^1^Institute of Advanced Materials (IAM), Nanjing Tech University (NanjingTech), 30 South Puzhu Road, Nanjing 211816, China; ^2^Frontiers Science Center for Flexible Electronics, Xi'an Institute of Flexible Electronics (IFE) and Xi'an Institute of Biomedical Materials & Engineering, Northwestern Polytechnical University, 127 West Youyi Road, Xi'an 710072, China; ^3^Key Laboratory for Organic Electronic & Information Displays (KLOEID) and Jiangsu Key Laboratory for Biosensors, Institute of Advanced Materials (IAM), Nanjing University of Posts and Telecommunications, 9 Wenyuan Road, Nanjing 210023, China

## Abstract

Most van der Waals two-dimensional (2D) materials without surface dangling bonds show limited surface activities except for their edge sites. Ultrathin Bi_2_Se_3_, a topological insulator that behaves metal-like under ambient conditions, has been overlooked on its surface activities. Herein, through a topochemical conversion process, ultrathin nanoporous Bi_2_Se_3_ layers were epitaxially deposited on BiOCl nanosheets with strong electronic coupling, leading to hybrid electronic states with further bandgap narrowing. Such oriented nanoporous Bi_2_Se_3_ layers possessed largely exposed active edge sites, along with improved surface roughness and film forming ability even on inkjet-printed flexible electrodes. Superior room-temperature NO_2_ sensing performance was achieved compared to other 2D materials under bent conditions. Our work demonstrates that creating nanoscale features in 2D materials through topochemical heteroepitaxy is promising to achieve both favorable electronic properties and surface activity toward practical applications.

## 1. Introduction

Bismuth-based two-dimensional (2D) layered materials, such as bismuth chalcogenides [1, 2], bismuth oxyhalides [3, 4], and bismuth halides [5, 6], are emerging eco-friendly functional materials that find wide applications in electronics, optoelectronics and energy conversion, and storage devices [7–9]. Bi_2_Se_3_ possesses excellent electrical conductivity even under a high density of defects and dislocations, since its surface states are protected from scattering [10–12]. Nanostructures of Bi_2_Se_3_ have recently shown potential in photothermal cancer therapy [13], optical switching [14], and thermoelectric devices [15]. In addition, they have also been combined with other nanostructures, such as graphene [16], ZnO [17], and CsPbBr_3_ [18], for enhanced photoabsorption, stimulated surface-plasmon polaritons, and enhanced exciton transfer efficiency, respectively. However, despite their outstanding electronic and optoelectronic properties, the surface activities of Bi_2_Se_3_ seem to be overlooked and less explored.

Bismuth oxyhalides (BiOX, where X = Cl, Br, and I) are particularly promising in photocatalytic energy conversion and environmental remediation [19–22]. BiOCl is perhaps one of the most investigated bismuth oxyhalides because of its low toxicity and high stability [23, 24]. However, its relatively large bandgap (typically ~3.2 eV) has limited its electronic and optoelectronic applications [22]. To date, many strategies have been developed to improve its properties, including morphology control [25], tuning of exposed crystal facets [21], and surface modification [26]. The construction of heterostructures, by considering band level alignment, has been proven effective in modifying its electronic properties, with examples including BiOCl/TiO_2_ [27], BiOCl/WO_3_ [28], BiOCl/ZnSn(OH)_6_ [29], and BiOCl/BiOI [30]. Epitaxial heterostructures are important with controllable overlayer orientation and coherent heterointerfaces [31]. Till now, solution-phased methods such as the coupling reaction [32] and self-assembly process [33] have been developed for preparing epitaxial heterostructures. However, the solution phase synthesis of BiOCl-based epitaxial heterostructures has been rarely explored.

Gas sensors are needed in many applications, including medical diagnosis [34], environmental monitoring [35], food quality assessment [36], and military applications [37]. Ultrathin 2D materials as well as their heterostructures have been applied to gas sensing due to large specific surface areas and tunable electronic and mechanical properties [38–41]. Many 2D materials, especially van der Waals 2D materials without surface dangling bonds, show higher activities at edges than at basal surfaces. However, maximizing edge exposure and increasing the edge/basal surface ratio remain great challenges.

In this study, we report the epitaxial growth of nanoporous Bi_2_Se_3_ with largely exposed edge sites on BiOCl nanosheets via an anion exchange-induced topochemical conversion process. Bi_2_Se_3_/BiOCl epitaxial heterostructures were used to detect NO_2_ gas and demonstrated good sensing performance even on printed flexible electrodes under bent conditions. This can be attributed to the strong electronic coupling across the Bi_2_Se_3_/BiOCl heterointerface, the enhanced surface activity of Bi_2_Se_3_, and the improved film forming ability with sub-2 nm surface features.

## 2. Results and Discussion

A solvothermal method was used to synthesize BiOCl nanosheets by following a previous report [22]. A solution of Se dissolved in oleylamine (OLA) and dodecanethiol (DDT) was then hot-injected into the BiOCl nanosheet solution and heated to induce the growth of Bi_2_Se_3_ on BiOCl.

**Figure 1:**
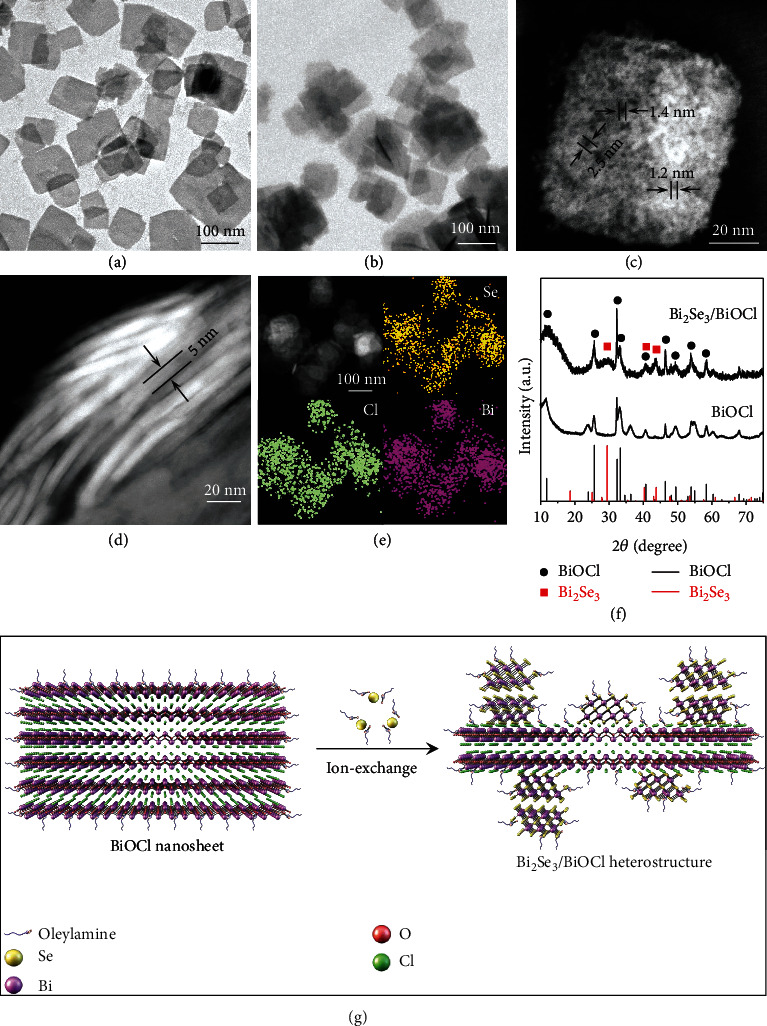
TEM images of (a) BiOCl nanosheets and (b) Bi_2_Se_3_/BiOCl heterostructures. (c) STEM image of a Bi_2_Se_3_/BiOCl heterostructure, revealing a nanoporous Bi_2_Se_3_ layer with a pore size of 1-3 nm. (d) Side-view STEM image of Bi_2_Se_3_/BiOCl heterostructures. (e) STEM image and EDX mapping of Bi_2_Se_3_/BiOCl heterostructures. (f) XRD patterns of BiOCl nanosheets and Bi_2_Se_3_/BiOCl heterostructures. (g) Schematic illustration of the formation process of the Bi_2_Se_3_/BiOCl heterostructure.

The BiOCl nanosheets and Bi_2_Se_3_/BiOCl heterostructures were characterized with transmission electron microscopy (TEM), energy dispersive X-ray spectroscopy (EDX), X-ray diffraction (XRD), and X-ray photoelectron spectroscopy (XPS), as shown in Figure 1 and Figure S1. The original BiOCl nanosheets were square-shaped with an average edge length of ~90 nm (Figure 1(a)) and thickness of 5-8 nm (Figure S1a). After being hybridized with Bi_2_Se_3_, the square shape of the BiOCl nanosheets remained, and their surfaces became fluffier (Figure 1(b)). The dark-field scanning transmission electron microscopy (STEM) images reveal that there are pores on the surface of the nanosheets, some of which are less than 2 nm in size (Figure 1(c) and Figure S1b, c), which was further proved by the pore size distribution curve extracted from the N_2_ adsorption-desorption isotherm curve (Figure S2). The thickness of the hybrid nanosheets, as estimated from their side-view STEM image, was ~5 nm (Figure 1(d)). Their EDX mapping analysis confirms the presence of Se, Cl, and Bi elements (Figure 1(e)), and the XRD pattern (Figure 1(f)) reveals peaks from both BiOCl (JCPDS no. 06-0249, space group *P*4*/nmm*, *a* = 0.3891 nm and *c* = 0.7369 nm) and Bi_2_Se_3_ (JCPDS no. 33-0214, space group *R*$\bar{3}$*m*, *a* = 0.4139 nm, and *c* = 2.8636 nm) [22, 42]. Their XPS Bi 4f spectrum shows two doublets for Bi^3+^ at 157.5/162.8 eV and 158.5/163.8 eV attributed to the different binding states of Bi in Bi_2_Se_3_ and BiOCl, respectively (Figure S3) [43, 44]. From the convoluted XPS peak areas, the Bi_2_Se_3_: BiOCl molar ratio can be estimated as 3 : 5 [45]. The formation of the Bi_2_Se_3_/BiOCl heterostructures is likely a result of an in situ ion-exchange reaction, as schematically shown in Figure 1(g). During this process, the Se powder dissolved in OLA and DDT was reduced to Se^2-^ and complexed with OLA based on Equation (1) [46]. The surface layer of BiOCl might undergo an ion exchange reaction with the surrounding Se^2-^ to produce Bi_2_Se_3_ (Equation (2)). This proposed in situ ion exchange process is also consistent with the observation that the Bi_2_Se_3_/BiOCl hybrid nanosheets did not become thicker than the original BiOCl nanosheets.

(1) $$\begin{array}{c} 2\text{OLA}+\text{Se}+2\text{HS}{\text{C}}_{15}{\text{H}}_{25}⟶{\left(\text{OLA}\right)}_{2}\text{Se}+{\text{H}}_{25}{\text{C}}_{12}{\text{S}}_{2}{\text{C}}_{12}{\text{H}}_{25}, \end{array}$$

(2) $$\begin{array}{c} 2\text{BiOCl}+3{\left(\text{OLA}\right)}_{2}\text{Se}⟶{\text{Bi}}_{2}{\text{Se}}_{3}+2{\left(\text{OLA}\right)}_{2}\text{O}+2\left(\text{OLA}\right)\text{Cl}. \end{array}$$

**Figure 2:**
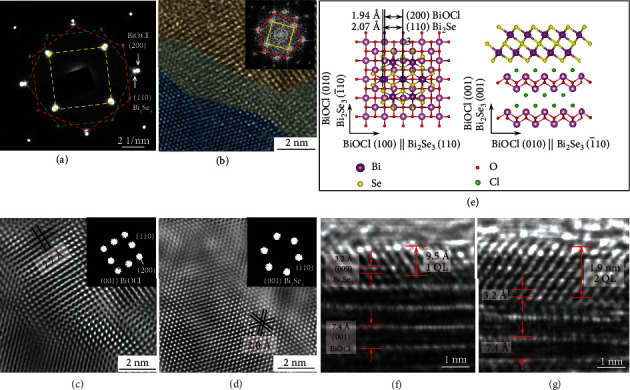
(a) SAED pattern of a Bi_2_Se_3_/BiOCl heterostructure. The pattern for BiOCl with a fourfold symmetry is indicated by a yellow square; two patterns for Bi_2_Se_3_ with a sixfold symmetry are indicated by red and green hexagons. (b) HRTEM image of an area with overlapping lattices of BiOCl and Bi_2_Se_3_. Inset: the corresponding FFT diffraction pattern. (c) BiOCl [001]-zone and (d) Bi_2_Se_3_ [001]-zone lattice patterns generated by performing inverse-FFT of the spots forming the red hexagon and the yellow square in (b), respectively. Insets in (c and d): the selected spots. (e) Schematic top-view and side-view models indicating the BiOCl (001) || Bi_2_Se_3_ (001) and BiOCl [100] || Bi_2_Se_3_ [110] epitaxial relationship. Side-view HRTEM images of typical Bi_2_Se_3_/BiOCl heterostructures with (f) 1 QL and (g) 2 QLs Bi_2_Se_3_ grown on a BiOCl nanosheet.

The microstructure of the obtained heterostructure was investigated with selected area electron diffraction (SAED) and high-resolution TEM (HRTEM), as shown in Figure 2. The SAED pattern shows three sets of patterns (Figure 2(a)). One has a fourfold symmetry (indicated by the yellow square) corresponding to BiOCl, and the other two have a sixfold symmetry (indicated by the red and green hexagons) corresponding to Bi_2_Se_3_. The presence of two hexagonal patterns suggests that the Bi_2_Se_3_ overlayer has two equivalent alignment directions with a relative rotation angle of 90^o^. In other words, the (110) planes of Bi_2_Se_3_ can be aligned with either the (200) or (020) planes of BiOCl, establishing an epitaxial relationship of BiOCl (001) || Bi_2_Se_3_ (001) and BiOCl [100] || Bi_2_Se_3_ [110]. In the HRTEM image in Figure 2(b), an overlap of lattice patterns of Bi_2_Se_3_ and BiOCl can be observed, and its fast Fourier transformation- (FFT-) generated diffraction pattern shows two sets of spots. By selecting the respective set of spots, the BiOCl [001]-zone and the Bi_2_Se_3_ [001]-zone lattice patterns were regenerated and are shown in Figures 2(c) and 2(d), respectively, further confirming the epitaxial relationship shown in Figure 2(e). The side-view HRTEM images of typical hybrid nanosheets show lattice spacings of 0.32 and 0.74 nm, attributable to the Bi_2_Se_3_ (009) and BiOCl (001) planes, respectively (Figures 2(f) and 2(g)). It can also be seen that the surface deposited Bi_2_Se_3_ layer contains one quintuple layer (QL) or two QLs.

**Figure 3:**
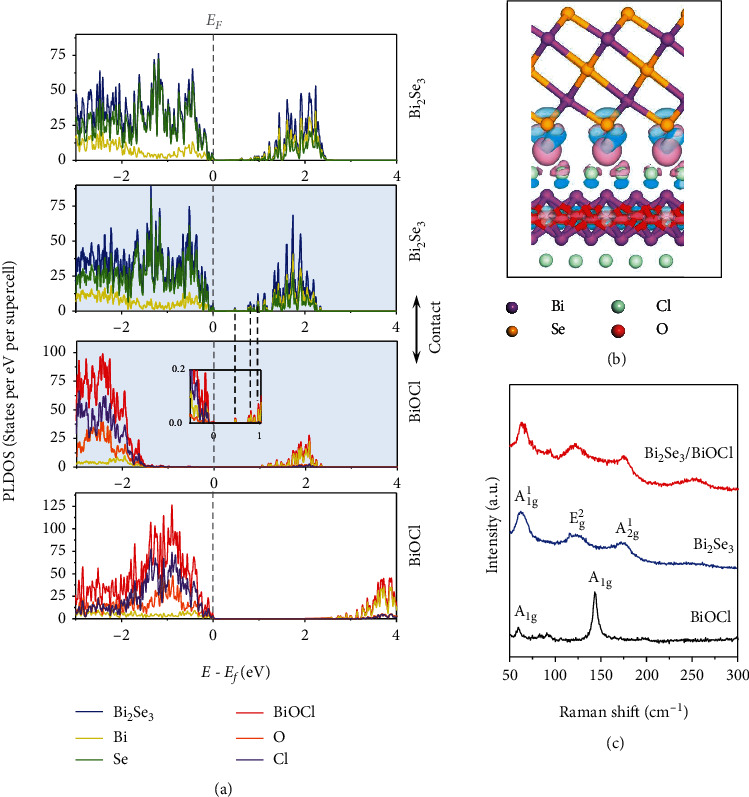
(a) Calculated PLDOS of Bi_2_Se_3_ (the top 1^st^ panel) and BiOCl (bottom panel) before and after (the 2^nd^ and 3^rd^ panels) contact. (b) Calculated structure model of the Bi_2_Se_3_-BiOCl interface superimposed with the charge density difference (cyan: positive; pink: negative). (c) Raman spectra of BiOCl, Bi_2_Se_3_, and Bi_2_Se_3_/BiOCl heterostructures.

The energy levels of BiOCl and Bi_2_Se_3_ (Figure S4, 5) were determined with UV-vis absorption spectra (Figure S6) and UV photoelectron spectroscopy (UPS, Figure S7). The results show that while Bi_2_Se_3_ shows n-type semiconducting behavior, BiOCl is a p-type semiconductor, consistent with previous reports [47, 48]. The electronic properties of the heterostructure were further studied with first-principle calculations based on density functional theory (DFT). A supercell based on 2 L Bi_2_Se_3_ and 2 L BiOCl was built, its geometric structure was optimized, and the projected local density of states (PLDOS) was calculated (Figures 3(a) and 3(b) and Figure S8). The Fermi level of BiOCl shifts closer to its conduction band edge after forming a heterojunction with Bi_2_Se_3_ (Figure 3(a)), suggesting electron transfer from Bi_2_Se_3_ to BiOCl across the interface. A zoomed-in PLDOS plot for BiOCl in the heterostructure (inset in the 3^rd^ panel in Figure 3(a)) shows new states that follow the projected states of Bi_2_Se_3_ (2^nd^ panel in Figure 3(a)), resulting in bandgap narrowing at the interface. The charge transfer across the interface can also be seen from the mapping of the charge density difference, in which electron deficient regions are observed on the Bi_2_Se_3_ side and electron rich regions appear toward the BiOCl side (Figure 3(b)). Such charge transfer across the interface and the change of the band structure of BiOCl were further demonstrated by the absence of the characteristic Raman peak for BiOCl (A_1g_ mode at 144 cm^−1^) in the Bi_2_Se_3_/BiOCl heterostructure (Figure 3(c)) [49, 50].

**Figure 4:**
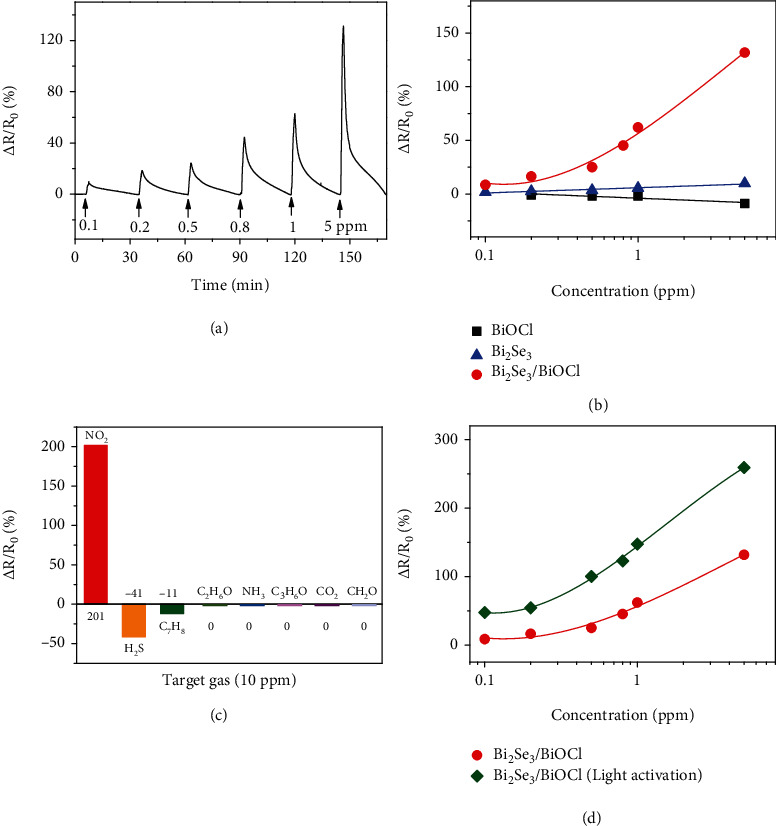
(a) Dynamic response-recovery curve of the sensor fabricated from Bi_2_Se_3_/BiOCl heterostructures in response to NO_2_ gas with increasing gas concentration at room temperature. (b) Sensing response vs. NO_2_ concentration for BiOCl-, Bi_2_Se_3_-, and Bi_2_Se_3_/BiOCl-based sensors. (c) Response of Bi_2_Se_3_/BiOCl heterostructures upon exposure to 10 ppm NO_2_, H_2_S, C_7_H_8_, C_2_H_5_OH, NH_3_, (CH_3_)_2_CO, CO_2_, and HCHO at room temperature. (d) Sensing response vs. NO_2_ concentration for Bi_2_Se_3_/BiOCl-based sensor with and without a 365 nm light irradiation (8.31 *μ*W).

The large specific surface area enabled by the formation of a nanoporous surface layer and the additional charge transfer channels enabled by the new electronic states upon heterojunction formation suggest that our epitaxial heterostructures are promising for sensing applications. As a proof-of-concept demonstration, the NO_2_ gas sensing properties of BiOCl nanosheets, Bi_2_Se_3_ nanosheets, and Bi_2_Se_3_/BiOCl heterostructures were studied at room temperature (Figures 4(a) and 4(b) and Figure S9). To preclude the influence of the baseline shift, baseline correction was implemented for the sensing responses (Figure S10) [51–53]. Upon exposure to NO_2_ (an oxidizing gas), the BiOCl film and Bi_2_Se_3_ film showed decreased and increased resistance, respectively, consistent with their *p*-type and *n*-type semiconducting properties [54–57]. However, their sensitivities are poor (e.g., 1.8% response at 1 ppm for BiOCl and 5.4% response at 1 ppm for Bi_2_Se_3_). The sensor based on Bi_2_Se_3_/BiOCl, which also showed an increased resistance upon NO_2_ exposure, exhibited ~35 and ~12 times higher responses than BiOCl and Bi_2_Se_3_ at 1 ppm, respectively (Figures 4(a) and 4(b)). In addition, the porous structure of the surface layer of the heterostructure resulted in a large specific surface area, which allowed more gas molecules to interact with the sensing material, but might cause a problem of slow gas desorption [58, 59]. This was also reflected in the slower recovery times than response times (Figure S11). The sensor was also capable of providing a 9.4% response at 100 ppb, along with good reproducibility (Figure S12). Its good selectivity toward NO_2_ was proven by exposing it to different gases at 10 ppm, including NO_2_, H_2_S, C_7_H_8_, C_2_H_5_OH, NH_3_, (CH_3_)_2_CO, CO_2_, and HCHO, at room temperature (Figure 4(c)). The sensing response of Bi_2_Se_3_/BiOCl could be further improved by light irradiation (Figure 4(d) and Figure S13). This is consistent with the charge transfer and carrier modulation-based sensing mechanism of this type of chemiresistive sensors.

**Figure 5:**
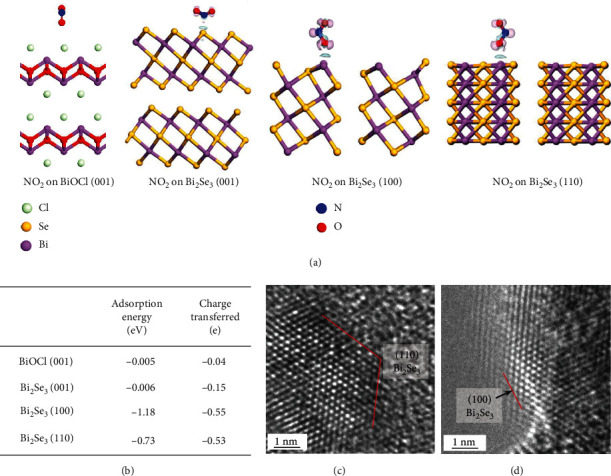
(a) Calculated structural models of NO_2_ molecules absorbed on the BiOCl (001), Bi_2_Se_3_ (001), (100), and (110) surfaces. (b) Calculated adsorption energy (eV) and electron transferred (e) toward NO_2_ on different sensing surfaces. HRTEM images showing the (c) (110) edge plane and (d) (100) edge plane of Bi_2_Se_3_.

The enhanced sensing performance of the Bi_2_Se_3_/BiOCl gas sensor at room temperature can be explained from the following aspects. First, since the sensing mechanism of our sensors is mainly based on charge transfer [60], DFT calculations were performed to shed light on how effective the NO_2_ adsorption can take away electrons from the heterostructures (Figure 5(a)). The adsorption energies of NO_2_ on the (110), (100), and (001) planes of Bi_2_Se_3_ were calculated to be -0.73 eV, -1.18 eV, and -0.006 eV, respectively, which are more negative than that on BiOCl (001) (-0.005 eV), indicating the higher affinity of NO_2_ toward Bi_2_Se_3_. Note that the edges of Bi_2_Se_3_ layers, i.e., the (110) and (100) facets are particularly more effective in NO_2_ adsorption and charge transfer compared to those on the (001) basal plane (Figure 5(b)). Indeed, the nanoporous Bi_2_Se_3_ layer epitaxially deposited on BiOCl was (001)-oriented, showing exposed (110) and (100) facets (Figures 5(c) and 5(d)), thus, favoring NO_2_ adsorption and electron transfer.

The improved sensing ability can also be attributed, in part, to the formation of a *p*-*n* junction at the epitaxial interface between BiOCl and Bi_2_Se_3_ (Figure S14), where the interdiffusion of electrons and holes across the interface created a charge depletion region along with a built-in potential barrier [61–64]. The height of the energy barrier was mainly determined by the hole concentration in BiOCl because of the much wider bandgap of BiOCl than that of Bi_2_Se_3_ [65, 66]. Exposure of Bi_2_Se_3_/BiOCl to NO_2_ gas increased the hole concentration in BiOCl, leading to a wider depletion region and thus a higher energy barrier. Because the conductance of the sensing material changes exponentially with the energy barrier at the heterojunction [67, 68], a much improved gas sensing response can be obtained. A higher carrier concentration in BiOCl could be achieved by light irradiation, as shown in Figure 4(d), where the sensing response of the Bi_2_Se_3_/BiOCl sensor doubled under 365 nm light excitation (8.31 *μ*W).

Moreover, the Bi_2_Se_3_/BiOCl heterostructure-based sensor showed a faster response time than both Bi_2_Se_3_ and BiOCl sensors (Figure S11). This can be attributed to the newly generated hybrid electronic states upon formation of epitaxial interface, as shown in the calculated PLDOS in Figure 3(a). These hybrid states could provide additional and faster channels to lose electrons to NO_2_, and therefore, a shorter response time was observed for the Bi_2_Se_3_/BiOCl-based sensing material.

**Figure 6:**
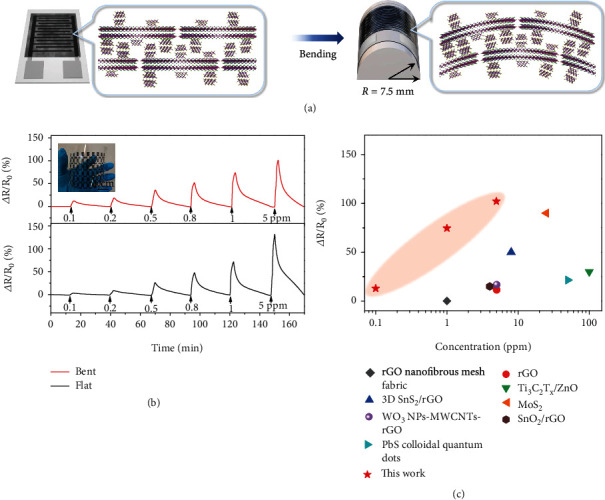
(a) Schematic diagram of the Bi_2_Se_3_/BiOCl heterostructures on flat and bent substrates. (b) Responses of Bi_2_Se_3_/BiOCl-based sensors under flat and bent states at various NO_2_ concentrations. Inset: a photograph of the printed electrode array. (c) Comparison of the sensing performance between our flexible gas sensor and other 2D material-based flexible sensors under bending conditions.

Last, flexible gas sensors based on Bi_2_Se_3_/BiOCl heterostructures were fabricated by depositing them on a plastic substrate with inkjet-printed interdigitated electrodes (Figure 6). It is worth mentioning that flexible electrodes fabricated with facile printing techniques such as inkjet printing suffer from poor resolution and large gaps between electrodes. This can cause reduced device performance compared to using electrodes fabricated by standard lithography techniques. The growth of the nanoporous Bi_2_Se_3_ layer on the BiOCl nanosheet increased the surface roughness and might help enhance the interlocking between the stacked hybrid nanosheets (Figure 6(a)). This ensured the formation of a continuous sensing film across the printed electrodes with a large gap of ~200 *μ*m (Figure S15), even though the average lateral size of the Bi_2_Se_3_/BiOCl nanosheets was less than 100 nm (Figure 1(b)). In sharp contrast, pristine BiOCl nanosheets deposited on inkjet-printed electrodes failed to form conductive films (Figure S16). The response of the flexible gas sensor under bent conditions (bending radius: 7.5 mm) showed a slightly enhanced response at sub-ppm levels compared to that under flat conditions, with a calculated limit of detection down to 1.6 ppb (Figure 6(b) and Figure S17), outperforming previously reported flexible sensors based on 2D materials under bent conditions (Figure 6(c) and Table S1) [69–75]. In addition, the bending radius also influenced the sensing performance. A further reduced bending radius to 5 mm could induce more exposed active edge sites and thus higher responses at sub-ppm levels (Figure S18). Last, the mechanical durability of our flexible sensors was tested by repeatedly bending a sensor for 100 times. The sensor maintained approximately 95% of its original response toward 5 ppm NO_2_ (Figure S19).

## 3. Conclusion

In this work, we demonstrated the in situ topochemical conversion of layered materials, which is capable of maximizing the active edge sites of the deposited overlayers and the formation of coherent heterointerfaces. Using Bi_2_Se_3_/BiOCl as a demonstration, onto BiOCl (001) basal surfaces with fourfold symmetry, hexagonal Bi_2_Se_3_ (001) layers were epitaxially deposited with largely exposed (100) and (110) edge sites. Compared to BiOCl or Bi_2_Se_3_ alone, the Bi_2_Se_3_/BiOCl heterostructure showed a much enhanced sensing response toward NO_2_ gas. According to the theoretical calculation results, the Bi_2_Se_3_ edge surfaces are generally more active than the basal surfaces of Bi_2_Se_3_ and BiOCl in NO_2_ adsorption and charge transfer. Flexible gas sensors based on Bi_2_Se_3_/BiOCl heterostructures possess good sensing properties with a limit of detection down to 1.6 ppb at room temperature, demonstrating their potential for wearable and portable devices in the future. Our strategy of generating nanopores in van der Waals layered materials via topochemical heteroepitaxy will provide more opportunities to tailor both their electronic and chemical properties.

## Data Availability

The data used to support the findings of this study are available from the corresponding authors upon request.

## Supplementary Materials

Supplementary Materials The detailed experimental methods on materials, synthesis of BiOCl nanosheets, synthesis of Bi2Se3 nanosheets, synthesis of Bi2Se3/BiOCl nanosheets, fabrication of chemiresistive sensors and gas sensing tests, fabrication of flexible sensors, characterization, and computational details, Figures S1-S19 and Table S1 are incorporated in the supplementary material.

## References

[1] Jin Q., Jiang S., Zhao Y. (2019). Flexible layer-structured Bi_2_Te_3_ thermoelectric on a carbon nanotube scaffold. *Nature Materials*.

[2] Yao J., Koski K. J., Luo W. (2014). Optical transmission enhacement through chemically tuned two-dimensional bismuth chalcogenide nanoplates. *Nature Communications*.

[3] Shi Y., Li J., Mao C. (2021). Van Der Waals gap-rich BiOCl atomic layers realizing efficient, pure-water CO_2_-to-CO photocatalysis. *Nature Communications*.

[4] Li M., Yu S., Huang H. (2019). Unprecedented eighteen-faceted BiOCl with a ternary facet junction boosting cascade charge flow and photo-redox. *Angewandte Chemie International Edition*.

[5] Dai Y., Poidevin C., Ochoa-Hernández C., Auer A. A., Tüysüz H. (2020). A supported bismuth halide perovskite photocatalyst for selective aliphatic and aromatic C–H bond activation. *Angewandte Chemie International Edition*.

[6] Yao L., Zeng Z., Cai C. (2021). Strong second- and third-harmonic generation in 1D chiral hybrid bismuth halides. *Journal of the American Chemical Society*.

[7] Chen Y., Tan C., Zhang H., Wang L. (2015). Two-dimensional graphene analogues for biomedical applications. *Chemical Society Reviews*.

[8] Zhang X., Cheng H., Zhang H. (2017). Recent progress in the preparation, assembly, transformation, and applications of layer-structured nanodisks beyond graphene. *Advanced Materials*.

[9] Zhai W., Xiong T., He Z. (2021). Nanodots derived from layered materials: synthesis and applications. *Advanced Materials*.

[10] Zhang H., Zhang X., Liu C., Lee S., Jie J. (2016). High-responsivity, high-detectivity, ultrafast topological insulator Bi_2_Se_3_/silicon heterostructure broadband photodetectors. *ACS Nano*.

[11] Zhang G., Qin H., Teng J. (2009). Quintuple-layer epitaxy of thin films of topological insulator Bi_2_Se_3_. *Applied Physics Letters*.

[12] Peng H., Lai K., Kong D. (2010). Aharonov-Bohm interference in topological insulator nanoribbons. *Nature Materials*.

[13] Xie H., Li Z., Sun Z. (2016). Metabolizable ultrathin Bi_2_Se_3_ nanosheets in imaging-guided photothermal therapy. *Small*.

[14] Hu Y., Tong M., Cheng X. A. (2021). Bi_2_Se_3_-functionalized metasurfaces for ultrafast all-optical switching and efficient modulation of terahertz waves. *ACS Photonics*.

[15] Xiong Y., Zhou G., Lai N.-C. (2021). Chemically switchable n-type and p-type conduction in bismuth selenide nanoribbons for thermoelectric energy harvesting. *ACS Nano*.

[16] Kim J., Park S., Jang H. (2017). Highly sensitive, gate-tunable, room-temperature mid-infrared photodetection based on graphene–Bi2Se3 heterostructure. *ACS Photonics*.

[17] Baitimirova M., Andzane J., Viter R. (2019). Improved crystalline structure and enhanced photoluminescence of ZnO nanolayers in Bi_2_Se_3_/ZnO heterostructures. *The Journal of Physical Chemistry C*.

[18] Tang Y., Jiang T., Zhou T. (2019). Ultrafast exciton transfer in perovskite CsPbBr3quantum dots and topological insulator Bi2Se3film heterostructure. *Nanotechnology*.

[19] Di J., Xia J., Li H., Guo S., Dai S. (2017). Bismuth oxyhalide layered materials for energy and environmental applications. *Nano Energy*.

[20] Wang S., Hai X., Ding X. (2017). Light-switchable oxygen vacancies in ultrafine Bi_5_O_7_Br nanotubes for boosting solar-driven nitrogen fixation in pure water. *Advanced Materials*.

[21] Jiang J., Zhao K., Xiao X., Zhang L. (2012). Synthesis and facet-dependent photoreactivity of BiOCl single-crystalline nanosheets. *Journal of the American Chemical Society*.

[22] Guan M., Xiao C., Zhang J. (2013). Vacancy associates promoting solar-driven photocatalytic activity of ultrathin bismuth oxychloride nanosheets. *Journal of the American Chemical Society*.

[23] Li J., Li H., Zhan G., Zhang L. (2017). Solar water splitting and nitrogen fixation with layered bismuth oxyhalides. *Accounts of Chemical Research*.

[24] Li J., Yu Y., Zhang L. (2014). Bismuth oxyhalide nanomaterials: layered structures meet photocatalysis. *Nanoscale*.

[25] Ouyang W., Su L., Fang X. (2018). UV photodetectors based on BiOCl nanosheet arrays: the effects of morphologies and electrode configurations. *Small*.

[26] Mi Y., Wen L., Wang Z. (2016). Fe(III) modified BiOCl ultrathin nanosheet towards high-efficient visible- light photocatalyst. *Nano Energy*.

[27] Ouyang W., Teng F., Fang X. (2018). High performance BiOCl nanosheets/TiO_2_ nanotube arrays heterojunction UV photodetector: the influences of self-induced inner electric fields in the BiOCl nanosheets. *Advanced Functional Materials*.

[28] Yang W., Wen Y., Zeng D. (2014). Interfacial charge transfer and enhanced photocatalytic performance for the heterojunction WO_3_/BiOCl: first-principles study. *Journal of Materials Chemistry A*.

[29] Wang H., Yuan X., Wu Y. (2017). Plasmonic Bi nanoparticles and BiOCl sheets as cocatalyst deposited on perovskite-type ZnSn(OH)_6_ microparticle with facet- oriented polyhedron for improved visible-light-driven photocatalysis. *Applied Catalysis B: Environmental*.

[30] Sun L., Xiang L., Zhao X. (2015). Enhanced visible-light photocatalytic activity of BiOI/BiOCl heterojunctions: key role of crystal facet combination. *ACS Catalysis*.

[31] Wang Y., Sun S., Zhang J., Huang Y. L., Chen W. (2021). Recent progress in epitaxial growth of two-dimensional phosphorus. *SmartMat*.

[32] Gao X., Zhu Y., Yi D. (2018). Ultrathin graphdiyne film on graphene through solution-phase van der Waals epitaxy. *Advances*.

[33] Shi E., Yuan B., Shiring S. B. (2020). Two-dimensional halide perovskite lateral epitaxial heterostructures. *Nature*.

[34] Peng G., Tisch U., Adams O. (2009). Diagnosing lung cancer in exhaled breath using gold nanoparticles. *Nature Nanotechnology*.

[35] Struzik M., Garbayo I., Pfenninger R., Rupp J. L. (2018). A simple and fast electrochemical CO2Sensor based on Li7La3Zr2O12for environmental monitoring. *Advanced Materials*.

[36] Zou Y., Zhou X., Zhu Y., Cheng X., Zhao D., Deng Y. (2019). sp^2^-hybridized carbon-containing block copolymer templated synthesis of mesoporous semiconducting metal oxides with excellent gas sensing property. *Accounts of Chemical Research*.

[37] Jeong S. Y., Kim J. S., Lee J. H. (2020). Rational design of semiconductor-based chemiresistors and their libraries for next-generation artificial olfaction. *Advanced Materials*.

[38] Wang X. S., Wang Z. W., Zhang J. D. (2018). Realization of vertical metal semiconductor heterostructures via solution phase epitaxy. *Nature Communications*.

[39] Wang Z., Jingjing Q., Wang X. (2018). Two-dimensional light-emitting materials: preparation, properties and applications. *Chemical Society Reviews*.

[40] Dai J., Ogbeide O., Macadam N. (2020). Printed gas sensors. *Chemical Society Reviews*.

[41] Yang K., Wang X., Li H. (2017). Composition- and phase-controlled synthesis and applications of alloyed phase heterostructures of transition metal disulphides. *Nanoscale*.

[42] Zhang Y., Zhang F., Xu Y. (2019). Epitaxial growth of topological insulators on semiconductors (Bi2Se3/Te@Se) toward high-performance photodetectors. *Methods*.

[43] Yang J., Wang C., Ju H. (2017). Integrated quasiplane heteronanostructures of MoSe_2_/Bi_2_Se_3_ hexagonal nanosheets: synergetic electrocatalytic water splitting and enhanced supercapacitor performance. *Advanced Functional Materials*.

[44] Jin J., Wang Y., He T. (2015). Preparation of thickness-tunable BiOCl nanosheets with high photocatalytic activity for photoreduction of CO_2_. *RSC Advances*.

[45] Wei D., Tian F., Lu Z., Yang H., Chen R. (2016). Facile synthesis of Ag/AgCl/BiOCl ternary nanocomposites for photocatalytic inactivation of *S. aureus* under visible light. *RSC Advances*.

[46] Liu Y., Yao D., Shen L., Zhang H., Zhang X., Yang B. (2012). Alkylthiol-enabled se powder dissolution in oleylamine at room temperature for the phosphine-free synthesis of copper-based quaternary selenide nanocrystals. *Journal of the American Chemical Society*.

[47] Zhang H., Alameen A., An X. (2019). Theoretical and experimental investigations of BiOCl for electrochemical adsorption of cesium ions. *Physical Chemistry Chemical Physics*.

[48] Zhang X., Li G., Fan C., Ding G., Wang Y., Han P. (2014). Theoretical insights into the adsorption of monatomic Ag on the (2 × 2) BiOCl (0 0 1) surfaces. *Computational Materials Science*.

[49] Osterhoudt G. B., Carelli R., Burch K. S., Katmis F., Gedik N., Moodera J. S. (2018). Charge transfer inEuS/Bi2Se3heterostructures as indicated by the absence of Raman scattering. *Physical Review B*.

[50] Bhattacharyya S., Akhgar G., Gebert M., Karel J., Edmonds M. T., Fuhrer M. S. (2021). Recent progress in proximity coupling of magnetism to topological insulators. *Advanced Materials*.

[51] Wu Z., Rong L., Yang J. (2021). Ion-conductive hydrogel-based stretchable, self-healing, and transparent NO_2_ sensor with high sensitivity and selectivity at room temperature. *Small*.

[52] Feuerstein D., Parker K. H., Boutelle M. G. (2009). Practical methods for noise removal: applications to spikes, nonstationary quasi-periodic noise, and baseline drift. *Analytical Chemistry*.

[53] Park C. H., Schroeder V., Kim B. J., Swager T. M. (2018). Ionic liquid-carbon nanotube sensor arrays for human breath related volatile organic compounds. *ACS Sensors*.

[54] Wu E., Xie Y., Yuan B. (2018). Ultrasensitive and fully reversible NO2Gas sensing based on p-type MoTe2under ultraviolet illumination. *ACS Sensors*.

[55] Wang J., Fatima-Ezzahra E., Dai J. (2022). Ligand-assisted deposition of ultra-small Au nanodots on Fe_2_O_3_/reduced graphene oxide for flexible gas sensors. *Nanoscale Advances*.

[56] Li S., Wang Z., Wang X. (2017). Orientation controlled preparation of nanoporous carbon nitride fibers and related composite for gas sensing under ambient conditions. *Nano Research*.

[57] Bao J., Zeng S., Dai J. (2021). Heterostructures between a tin-based intermetallic compound and a layered semiconductor for gas sensing. *Chemical Communications*.

[58] Zhao L., Guanhua N., Lulu S. (2020). Effect of ionic liquid treatment on pore structure and fractal characteristics of low rank coal. *Fuel*.

[59] Yu Q., Zhu J., Xu Z., Huang X. (2015). Facile synthesis of *α*-Fe_2_O_3_@SnO_2_ core-shell heterostructure nanotubes for high performance gas sensors. *Sensors and Actuators B: Chemical*.

[60] Kim J.-S., Yoo H.-W., Choi H. O., Jung H.-T. (2014). Tunable volatile organic compounds sensor by using thiolated ligand conjugation on MoS_2_. *Nano Letters*.

[61] Ju D., Xu H., Qiu Z., Guo J., Zhang J., Cao B. (2014). Highly sensitive and selective triethylamine-sensing properties of nanosheets directly grown on ceramic tube by forming NiO/ZnO PN heterojunction. *Sensors and Actuators B: Chemical*.

[62] Sharma B., Sharma A., Myung J.-H. (2021). Selective ppb-level NO_2_ gas sensor based on SnO_2_-boron nitride nanotubes. *Sensors and Actuators B: Chemical*.

[63] Zheng W., Xu Y., Zheng L. (2020). MoS2 Van der Waals p–n junctions enabling highly selective room-temperature NO2 sensor. *Advanced Functional Materials*.

[64] Cao J., Chen Q., Wang X. (2021). Recent development of gas sensing platforms based on 2D atomic crystals. *Research*.

[65] Cui G., Zhang M., Zou G. (2013). Resonant tunneling modulation in quasi-2D Cu_2_O/SnO_2_ p-n horizontal-multi- layer heterostructure for room temperature H_2_S sensor application. *Scientific Reports*.

[66] Jiang L., Xue K., Chen Z., Cui Q., Xu S. (2022). High performance of gas sensor based on Bi-doped ZnSnO_3_/CuO nanocomposites for acetone. *Microporous and Mesoporous Materials*.

[67] Hu Y., Zhou J., Yeh P.-H., Li Z., Wei T. Y., Wang Z. L. (2010). Supersensitive, fast-response nanowire sensors by using Schottky contacts. *Advanced Materials*.

[68] Choi J., Kim Y.-J., Cho S.-Y. (2020). In situ formation of multiple Schottky barriers in a Ti_3_C_2_ MXene film and its application in highly sensitive gas sensors. *Advanced Functional Materials*.

[69] Park H. J., Kim W.-J., Lee H.-K. (2018). Highly flexible, mechanically stable, and sensitive NO_2_ gas sensors based on reduced graphene oxide nanofibrous mesh fabric for flexible electronics. *Sensors and Actuators B: Chemical*.

[70] Su P.-G., Shieh H.-C. (2014). Flexible NO_2_ sensors fabricated by layer-by-layer covalent anchoring and in situ reduction of graphene oxide. *Sensors and Actuators B: Chemical*.

[71] Wu J., Wu Z., Ding H. (2020). Flexible, 3D SnS_2_/reduced graphene oxide heterostructured NO_2_ sensor. *Sensors and Actuators B: Chemical*.

[72] Yang Z., Jiang L., Wang J. (2021). Flexible resistive NO_2_ gas sensor of three-dimensional crumpled MXene Ti_3_C_2_T_x_/ZnO spheres for room temperature application. *Sensors and Actuators B: Chemical*.

[73] Yaqoob U., Uddin A. S. M. I., Chung G.-S. (2016). A high-performance flexible NO_2_ sensor based on WO_3_ NPs decorated on MWCNTs and RGO hybrids on PI/PET substrates. *Sensors and Actuators B: Chemical*.

[74] Liu H., Li M., Voznyy O. (2014). Physically flexible, rapid-response gas sensor based on colloidal quantum dot solids. *Advanced Materials*.

[75] Wu J., Wu Z., Ding H. (2020). Three-dimensional graphene hydrogel decorated with SnO2 for high-performance NO2 sensing with enhanced immunity to humidity. *ACS Applied Materials & Interfaces*.

