# Supplementary material for: Realization of Oriented and Nanoporous Bismuth Chalcogenide Layers via Topochemical Heteroepitaxy for Flexible Gas Sensors
Source: Research (Wash D C). 2022 Jun 23;2022:9767651. doi: 10.34133/2022/9767651 (PMC9275095; doi:10.34133/2022/9767651)
Supplement: Supplementary Materials — The detailed experimental methods on materials, synthesis of BiOCl nanosheets, synthesis of Bi2Se3 nanosheets, synthesis of Bi2Se3/BiOCl nanosheets, fabrication of chemiresistive sensors and gas sensing tests, fabrication of flexible sensors, characterization, and computational details, Figures S1-S19 and Table S1 are incorporated in the supplementary material. [file 9767651.f1.docx]

**Supporting Information**

Realization of Oriented and Nanoporous Bismuth Chalcogenide Layers via Topochemical Heteroepitaxy for Flexible Gas Sensors

Zhiwei Wang,^1, 2^ Jie Dai,^1^ Jian Wang,^1^ Xinzhe Li,^1^ Chengjie Pei,^1^ Yanlei Liu,^1^ Jiaxu Yan,^1^ Lin Wang,^1^ Shaozhou Li,^3^ Hai Li,^1^ Xiaoshan Wang,* ^2^ Xiao Huang,* ^1^ Wei Huang,* ^1, 2, 3^

^1^ Institute of Advanced Materials (IAM), Nanjing Tech University (NanjingTech), 30 South Puzhu Road, Nanjing 211816, China

^2^ Frontiers Science Center for Flexible Electronics, Xi'an Institute of Flexible Electronics (IFE) and Xi'an Institute of Biomedical Materials & Engineering, Northwestern Polytechnical University, 127 West Youyi Road, Xi’an 710072, China

^3^ Key Laboratory for Organic Electronic & Information Displays (KLOEID) and Jiangsu Key Laboratory for Biosensors, Institute of Advanced Materials (IAM), Nanjing University of Posts and Telecommunications, 9 Wenyuan Road, Nanjing 210023, China;

Correspondence should be addressed to Xiaoshan Wang, iamxswang@nwpu.edu.cn; Xiao Huang, iamxhuang@njtech.edu.cn; and Wei Huang, [iamwhuang@nwpu.edu.cn](mailto:iamwhuang@nwpu.edu.cn)

**EXPERIMENTAL PROCEDURES**

**Materials**

Bismuth nitrate pentahydrate (Bi(NO_3_)_3_·5H_2_O, ACS reagent, ≥98.0%, Sigma-Aldrich), mannitol (C_6_H_14_O_6_, 99%, Alfa Aesar), sodium chloride (NaCl, ≥99.8%, Sinopharm Chemical Reagent Co., Ltd.), polyvinyl pyrrolidone (PVP, Mw=10000, Macklin), selenium powder (100 mesh, 99.99%, Sigma-Aldrich), oleylamine (OLA, technical grade, 70%, Sigma-Aldrich), 1-dodecanethiol (DDT, 98%, Aladdin), ethanol (C_2_H_6_O, 99.7%, Sinopharm Chemical Reagent Co., Ltd.).

**Synthesis of BiOCl Nanosheets**

In a typical experiment, 1 mmol of Bi(NO_3_)_3_·5H_2_O and 0.4 g PVP were put into a 50 mL round-bottom flask that contained 25 mL 0.1 M mannitol solution (0.455 g mannitol in 25 mL H_2_O). The mixture was stirred and sonicated until Bi(NO_3_)_3_·5H_2_O was dissolved, followed by the dropwise injection of 5 mL saturated sodium chloride solution, resulting in the formation of a uniform white suspension. Then, the mixture was transferred to a Teflon-lined stainless-steel autoclave to perform the hydrothermal process at 160 °C for 3 h. After cooling to room temperature, the solid product was collected by centrifugation at 6,000 rpm for 5 min and washed with deionized water two times and ethanol two times to remove any possible remaining impurity. The samples were finally dried in a vacuum oven for 12 h at 40 °C for further synthesis and characterization.

**Synthesis of Bi_2_Se_3_ Nanosheets**

NaBH_4_ was reacted with Se powders with a molar ratio of 2:1 (1.334 mol L^-1^ : 0.667 mol L^-1^) in an ice-water bath under Ar for 30 min to form NaHSe aqueous solution. Then, 0.5 g PVP and 0.226 g Bi(NO_3_)_3_·5H_2_O were dissolved in 32.5 mL of ethylene glycol (EG) under magnetic stirring at room temperature. The flask was sealed and heated to 160 °C under Ar until the solution turned turbid. The freshly synthesized oxygen-free NaHSe solution (0.667 mol L^-1^, 1.048 mL) was rapidly injected into the mixture by a syringe. The reaction was cooled to room temperature 10 min later. The samples were collected by centrifugation at 12,000 rpm for 10 min and washed with with a mixture of acetone (400 mL) and ultrapure water (80 mL) three times.

**Synthesis of Bi_2_Se_3_/BiOCl Nanosheets**

BiOCl (0.3 mmol) was added to 10 mL OLA in a three-necked flask (100 mL) at room temperature. Then, the solution was vacuumed at 120 °C to remove the water and oxygen. About 5 min later, the solution was purged with argon. To this solution, the Se dispersion formed by oscillation of 0.3 mmol Se powder in a mixture of 1 mL OLA and 0.5 mL DDT at room temperature was quickly injected at 120 °C. After the resulting mixture was stirred at 120 °C for 3 h, it was cooled down to room temperature. The obtained products were collected by centrifugation at 7,000 rpm. for 5 min. To completely remove the residual Se precursor (easily oxidized to form Se powder), the precipitate was first washed with a mixture of 3 mL OLA and 0.2 mL DDT for one time, and then washed with a mixture of toluene and ethanol for two times. Finally, the Bi_2_Se_3_/BiOCl nanosheets were redispersed in ethanol.

**Fabrication of Chemiresistive Sensors and Gas Sensing Tests**

Chemiresistive gas sensors were fabricated based on the as-prepared materials for sensing various gases, including NO_2_, H_2_S, C_7_H_8_, C_2_H_5_OH, NH_3_, (CH_3_)_2_CO, CO_2_ and HCHO. Typically, several drops of the as-prepared product solutions were spin-cast onto an interdigitated Au-electrode (Au IDE, with 0.1 mm spacing over a 2 × 1 cm^2^ area, Changchun Mega Borui Technology Co., Ltd) and then dried in a vacuum oven at room temperature. The gas sensing test was performed in an airtight chamber with electrical feedthroughs at room temperature (25 °C). A constant current was applied to the sensor electrode, and the variation in the sensor resistance was monitored and recorded with the changes in the gas environment using a data acquisition system (34972A, Agilent) with a 20-channel multiplexer (34901A, Agilent). The response of the sensor is defined by its resistance variation ΔR/R_0_ (%) (ΔR = R_g_ - R_0_, where R_g_ and R_0_ are the resistance of the sample in the target gas and pure N_2_, respectively).

**Fabrication of flexible sensors**

The sensor devices were fabricated on 0.1 mm-thick flexible polyethylene terephthalate (PET) substrates by using an inkjet printer (Prtronic Scientific 3, China). The Ag ink purchased from Shanghai Mifang Electronic Technology Co., Ltd was inkjet-printed on the PET substrate to form an array of interdigitated Ag electrodes (0.2 mm spacing over a 1.5 × 1 cm^2^ area). Then, several drops of ethanol dispersion of Bi_2_Se_3_/BiOCl were spin-cast onto the Ag electrode.

**Characterization**

The X-ray photoelectron spectroscopy (XPS) and X-ray diffraction patterns (XRD) data were obtained using a Thermo ESCALAB 250XI (America) and Rigaku SmartLab diffractometer (Janpan) with Cu Kα radiation (λ = 1.54 Å), respectively. Samples for transmission electron microscopy (TEM) analysis were prepared by drying a drop of nanocrystal dispersion in ethanol on amorphous carbon-coated copper grids. TEM and high-resolution TEM (HRTEM) characterization were performed with a JEOL 2100Plus (Japan) and a JEOL 2100F (Japan) operated at 200 kV. The Ultraviolet photoelectron spectroscopy (UPS, VersaProbe. PHI 5000, Japan) and ultraviolet-vis absorption spectra (UV-vis, Shimadzu, UV-1750, Japan) were used to measure the electronic work functions and band levels.

**Computational Details**

First-principles calculations based on density functional theory (DFT) were performed with the Cambridge Serial Total Energy Package (CASTEP) in Materials Studio of Accelrys Inc [[1](#_ENREF_1)]. Generalized Gradient Approximation (GGA) of the Perdew-Burke-Ernzerhof (PBE) method was used in the approximation of the exchange-correlation part [[2](#_ENREF_2), [3](#_ENREF_3)]. For density of states (DOS) calculations of the heterostructures, a supercell containing 2 layers of BiOCl and 2 layers of Bi_2_Se_3_ was built. An ultrasoft pseudopotential was applied and the cut-oﬀ energy was set to 500 eV. The Brilloiouinzone k-point sampling was performed in a 7 × 7 × 1 Monkhorst-Pack scheme. The convergence tolerance criteria for the self-consistent ﬁeld, energy, maximum force, maximum stress, and maximum displacement were set to 1.0 × 10^-6^ eV atom^-1^, 1.0 × 10^-5^ eV atom^-1^, 0.03 eV A^-1^, 0.01 GPa and 0.001 A.


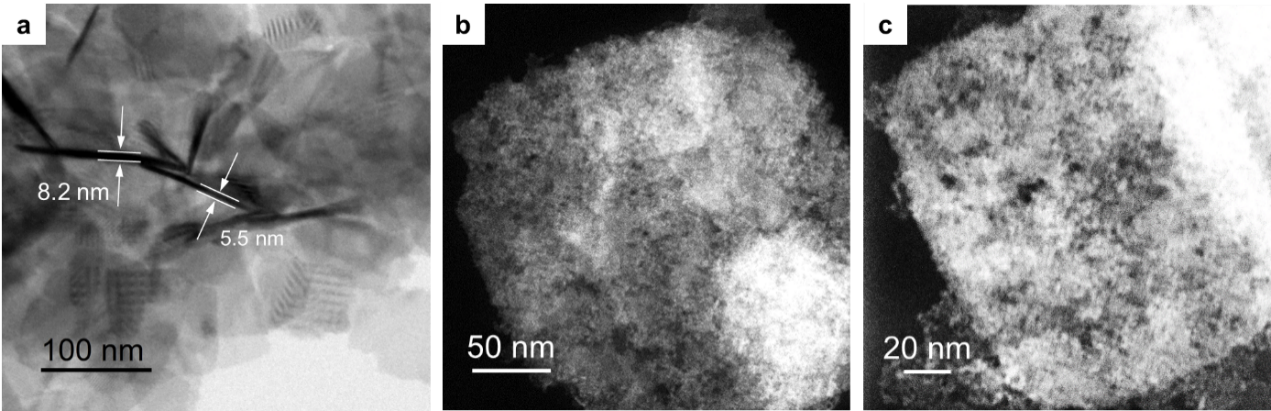


**Figure S1** a) TEM image of the BiOCl nanosheets. b) and c) STEM images of nanoporous Bi_2_Se_3_ layers grown on BiOCl nanosheets.


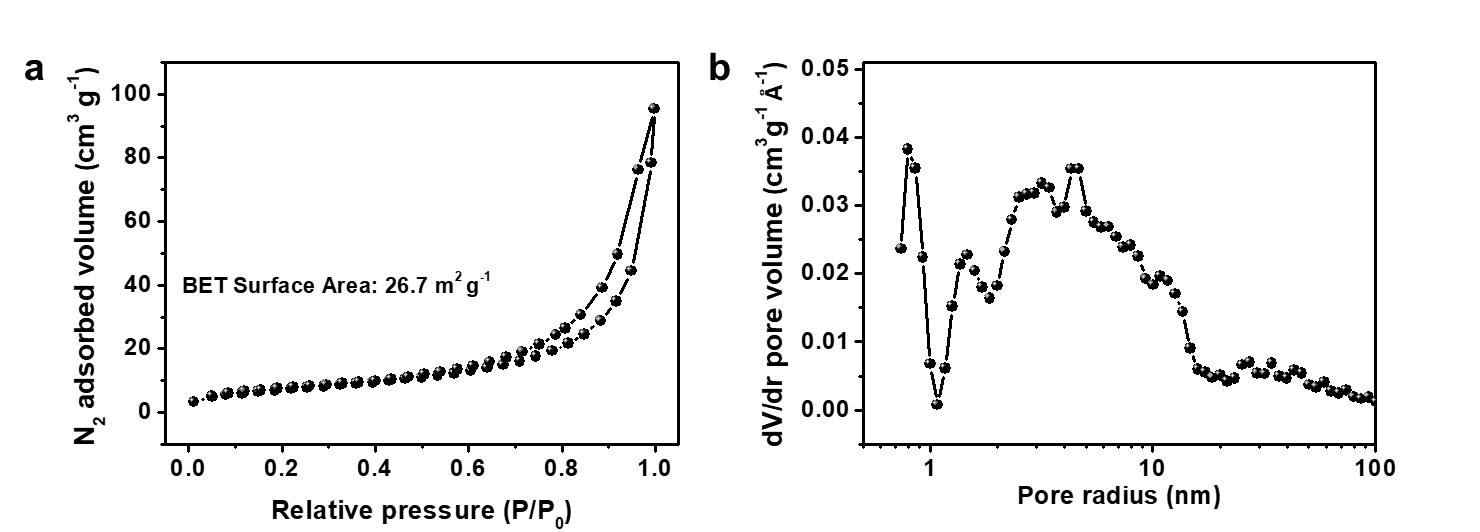


**Figure S2** a) N_2_ adsorption-desorption isotherm and b) DFT pore size distribution plot for Bi_2_Se_3_/BiOCl heterostructures.


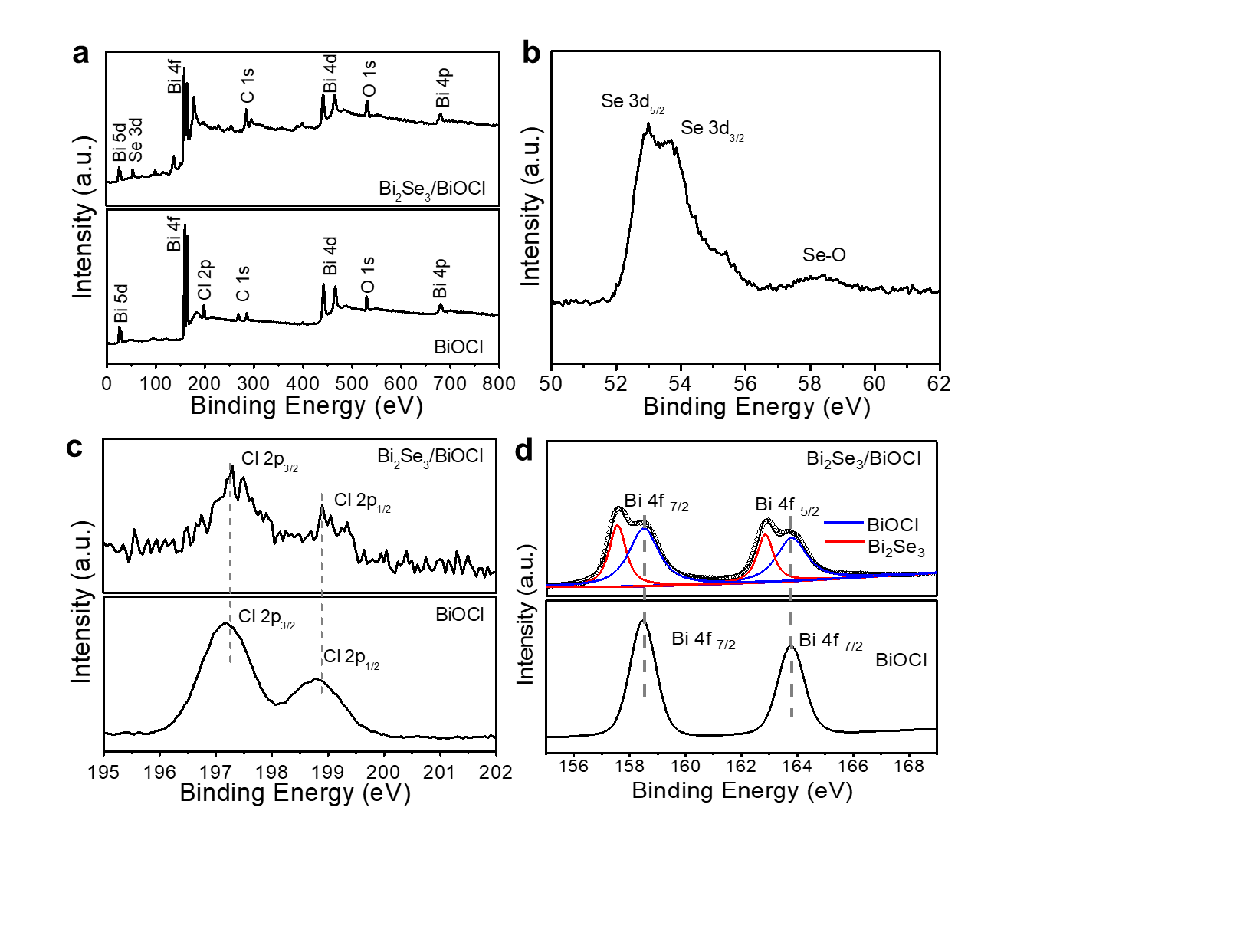


**Figure S3** a) Survey and high-resolution b) Se 3d, c) Cl 2p and d) Bi 4f spectra of Bi_2_Se_3_/BiOCl heterostructures and BiOCl nanosheets.

XPS analysis was used to determine the chemical states and surface composition of the as-synthesized BiOCl nanosheets and Bi_2_Se_3_/BiOCl heterostructures. The XPS spectra were corrected by referencing the C 1s peak of 284.60 eV. The peaks of Bi, O, Cl, Se and C elements can be well identified in the survey spectra, as shown in Figure S2a. In the Se 3d spectrum (Figure S2b), an extra band between 58 and 60 eV that corresponds to the surface oxidation is observed which suggests that a part of the surface Bi_2_Se_3_ samples may be oxidized into bismuth selenite [[4](#_ENREF_4)]. The peaks at 196.5 eV and 197.6 eV can be assigned to Cl 2p_3/2_ and 2p_1/2_ (Figure S2c), respectively.


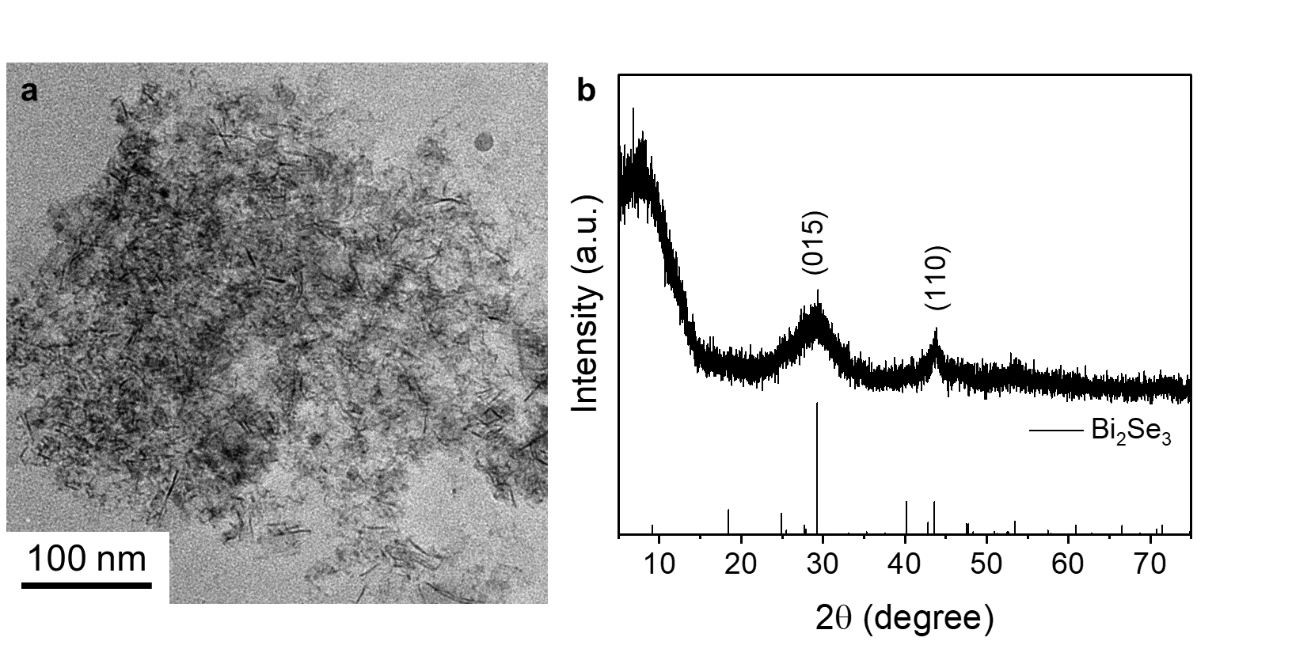


**Figure S4** a) TEM image and b) XRD pattern of Bi_2_Se_3_ nanosheets.


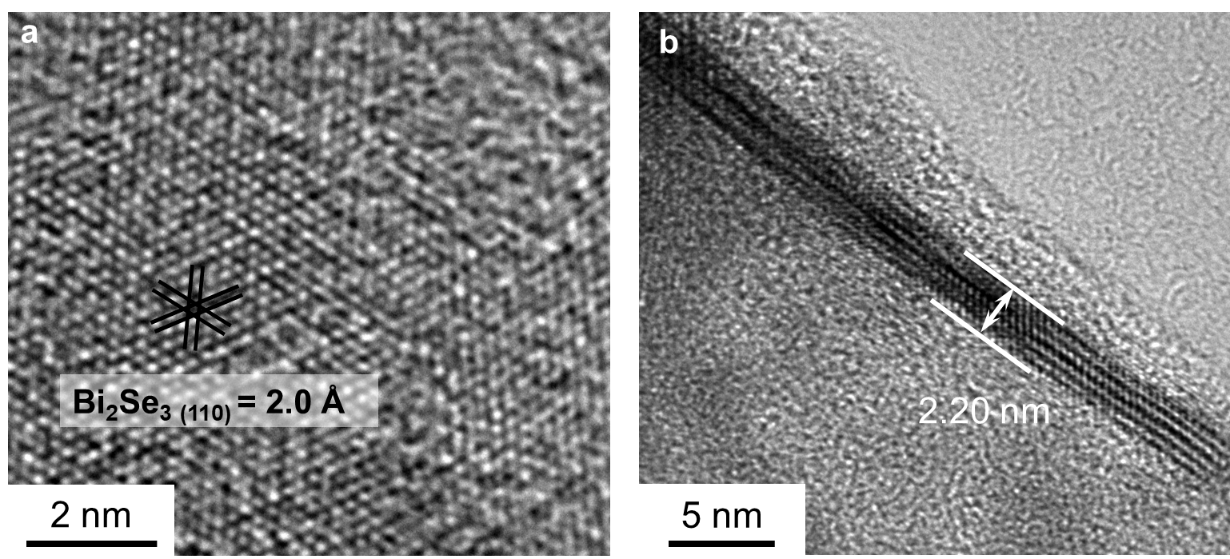


**Figure S5** a) Top-view and b) Side-view HRTEM images of Bi_2_Se_3_ nanosheets.

**
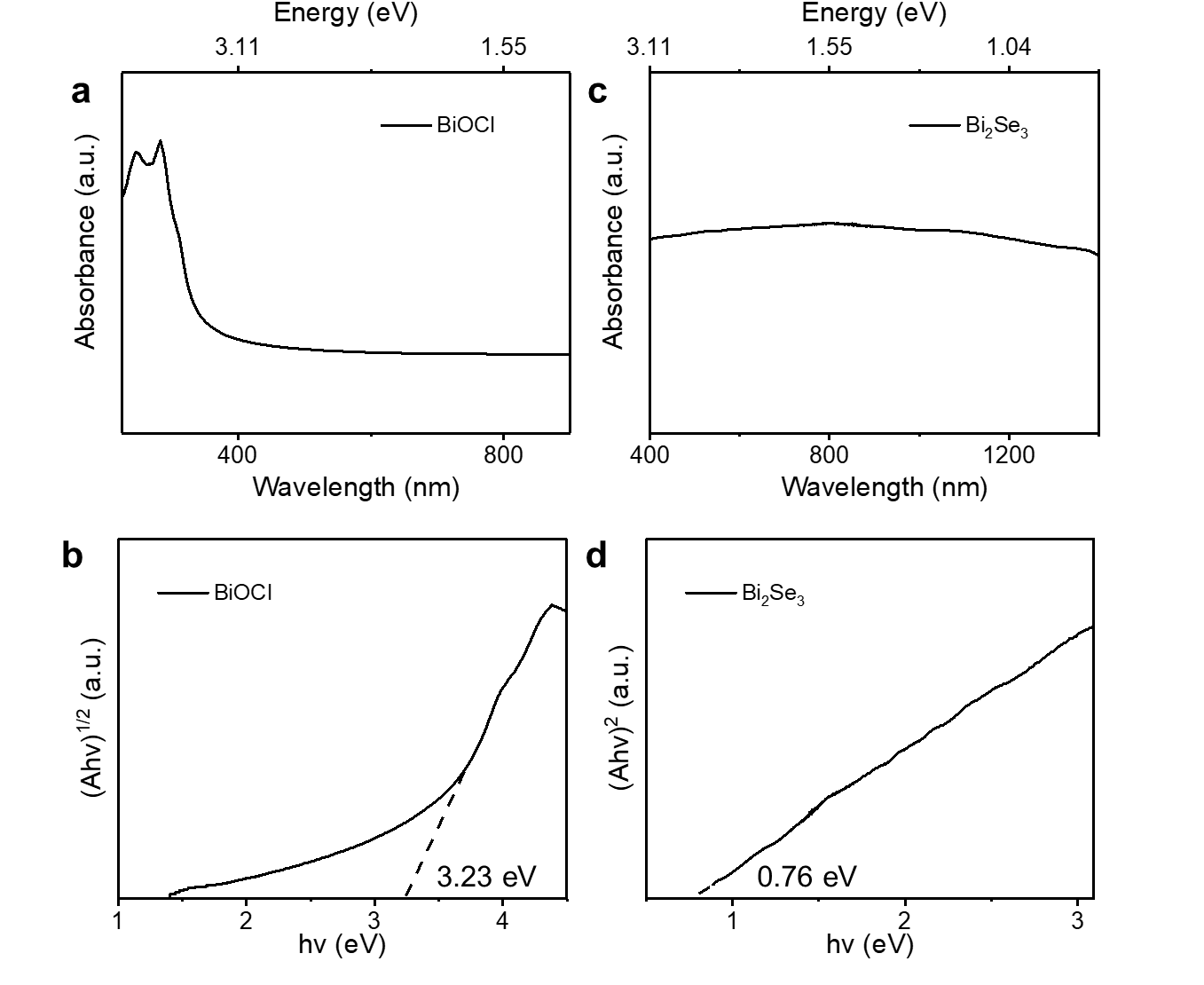
**

**Figure S6** UV-vis absorption spectra of a) BiOCl nanosheets and c) Bi_2_Se_3_ nanosheets. The bandgap values of b) the BiOCl nanosheets and d) Bi_2_Se_3_ nanosheets were estimated by a related curve of (*αhν*)^1/2^ and (*αhν*)^2^ versus photon energy plotted. It shows that pure BiOCl exhibits an absorption edge in the UV region and its bandgap is ∼3.23 eV. The Bi_2_Se_3_ sheets show wider absorption between 400 and 1400 nm and a narrow bandgap of 0.76 eV [[5](#_ENREF_5), [6](#_ENREF_6)].


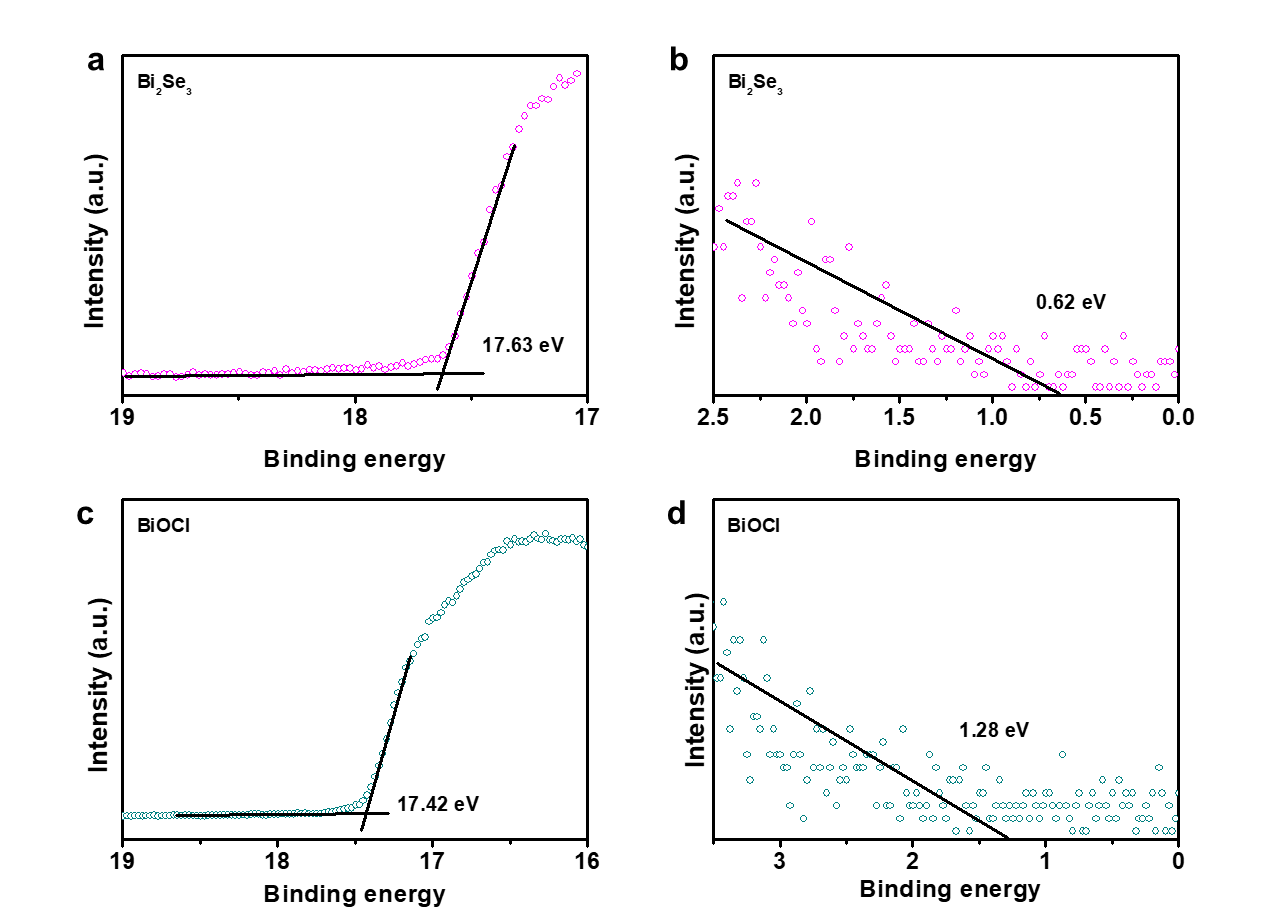


**Figure S7** Zoomed-in UPS spectra showing the positions of the Fermi level relative to the vacuum level for a) Bi_2_Se_3_ and c) BiOCl, and the positions of the valence band maximum (VBM) relative to the Fermi level (set to 0) for b) Bi_2_Se_3_ and d) BiOCl.


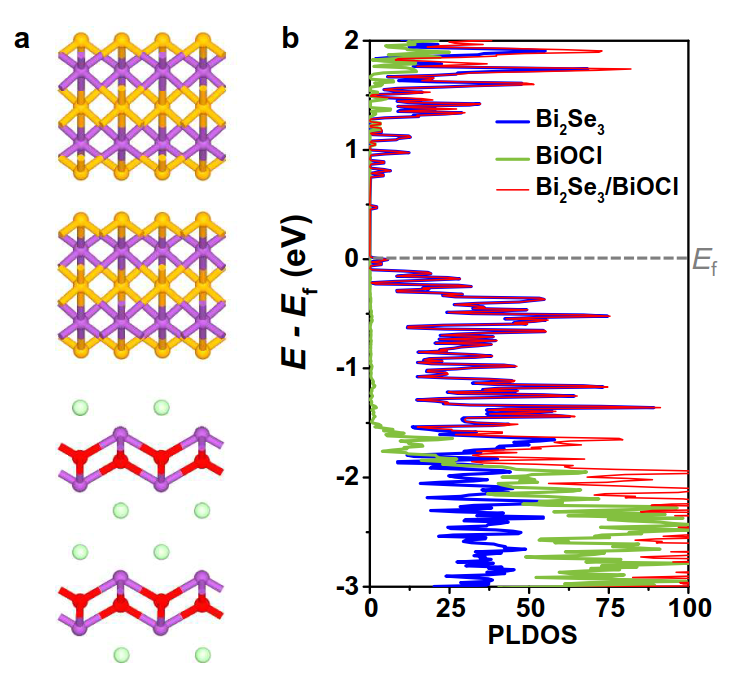


**Figure S8** a) Calculated structure model and b) projected local density of states (PLDOS) of a heterostructure containing 2L Bi_2_Se_3_ and 2L BiOCl. The DOS of the heterostructure near the band edges was mainly contributed by Bi_2_Se_3_.


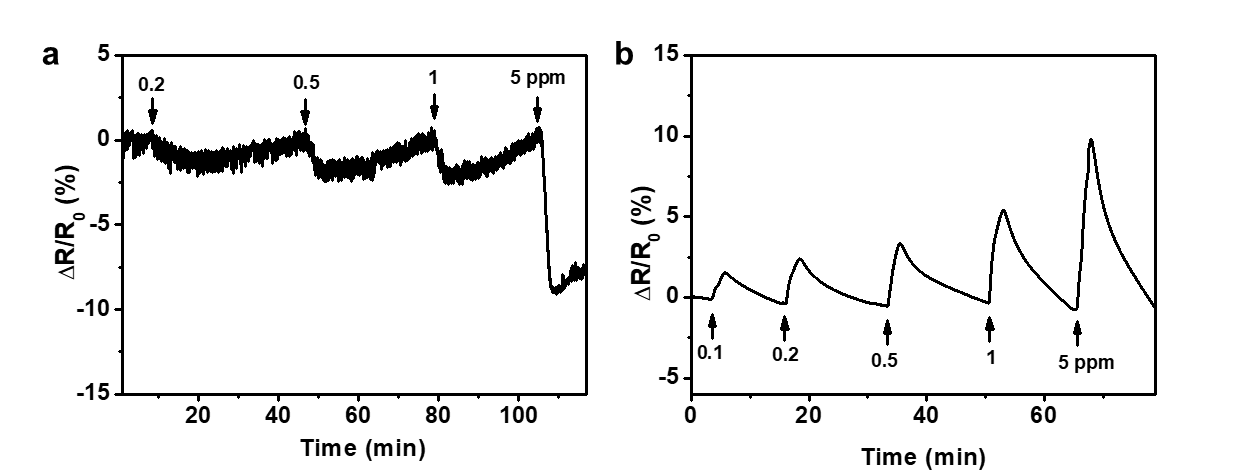


**Figure S9** Dynamic response-recovery curves of sensors fabricated from a) BiOCl nanosheets and b) Bi_2_Se_3_ nanosheets.


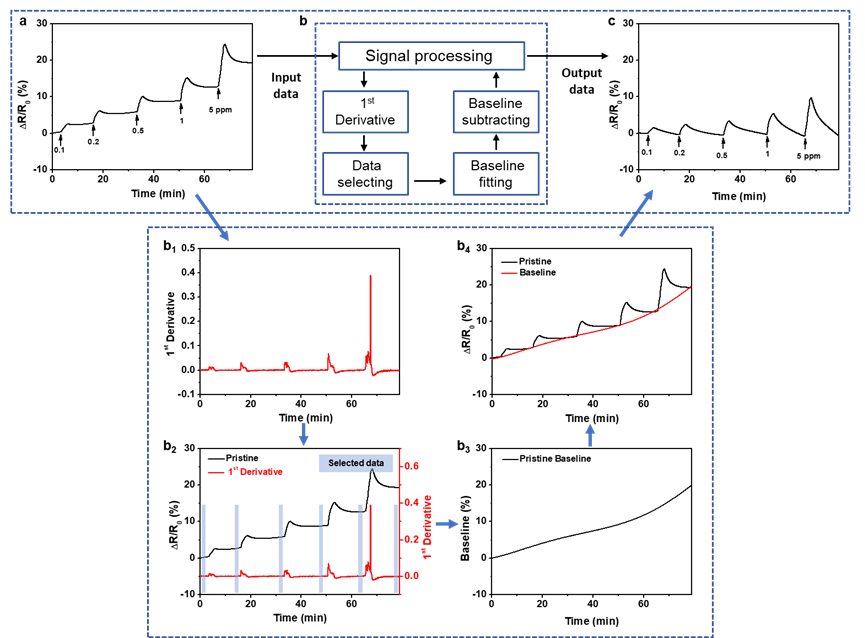


**Figure S10** a) The original response of Bi_2_Se_3_ nanosheet-based sensor to NO2 before baseline correction. b) The procedure of baseline correction: b1) The plot of 1^st^ derivative of Bi_2_Se_3_ nanosheets in response to NO2; b2) The plot of the dynamic response curve and its 1^st^ derivative of Bi_2_Se_3_ nanosheets in response to NO2; b3) The corrected baseline of dynamic response; b4) The baseline correction of the dynamic response to NO2. c) The plot of corrected response of Bi_2_Se_3_ nanosheet-based sensor to NO2.

The detailed procedure for baseline correction is as follow (Figure S10). First, the first order derivative of the original response data was obtained (b_1_). Second, the original response data which were located at the recovery period and meanwhile corresponding to the first order derivative equal to 0 were selected (marked in blue in b_2_), and then fitted to the baseline (b_3_). Then, the corrected response was obtained by subtracting the corrected baseline from the dynamic response curve (b_4_).


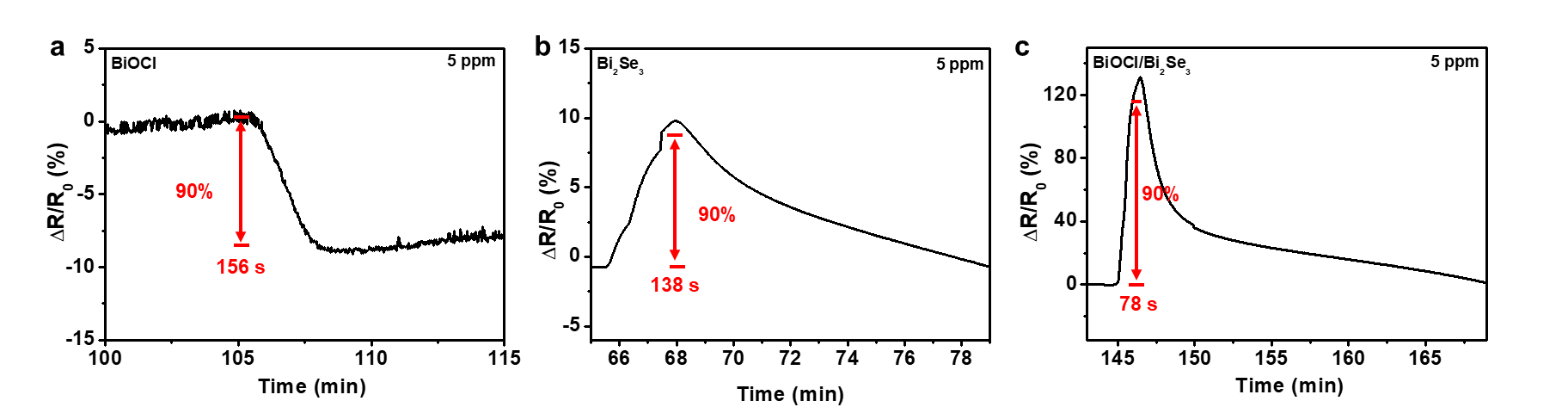


**Figure S11** Response time of (a) BiOCl, (b) Bi_2_Se_3_ and (c) BiOCl/Bi_2_Se_3_ to 5 ppm NO_2_.


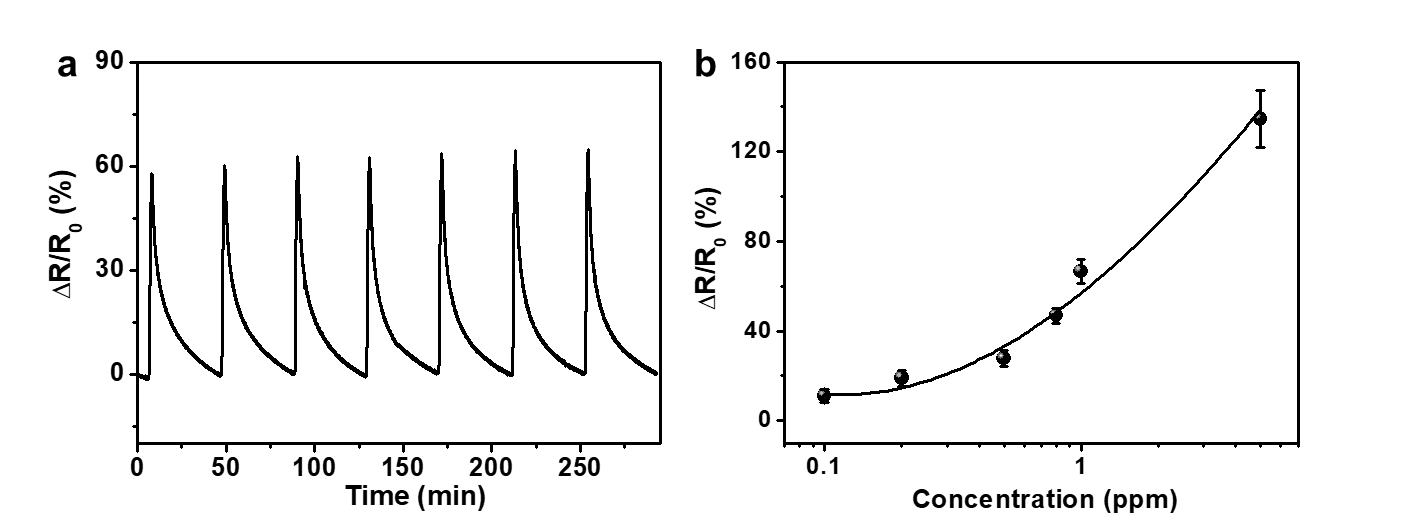


**Figure S12** a) Response changes of Bi_2_Se_3_/BiOCl heterostructures during seven successive cycles of exposure to 1 ppm NO_2_ gas. b) Response of Bi_2_Se_3_/BiOCl heterostructures upon exposure to NO_2_ with varied concentrations. Each error bar indicates the standard deviation of the sensing response for 5 experimental replicates.

**Figure S13** Dynamic response-recovery curve of the BiOCl/Bi_2_Se_3_ sensor to NO_2_ under a 365 nm light irradiation (8.31 μW).


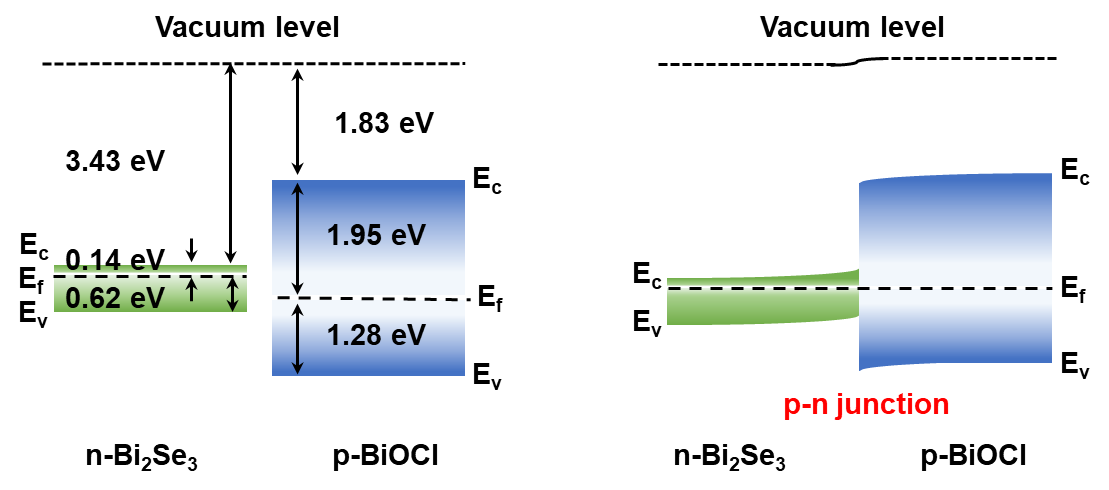


**Figure S14** Schematic diagram of the band level alignment diagram of Bi_2_Se_3_/BiOCl heterostructures.

**Figure S15** Optical microscopy image of the printed electrodes.


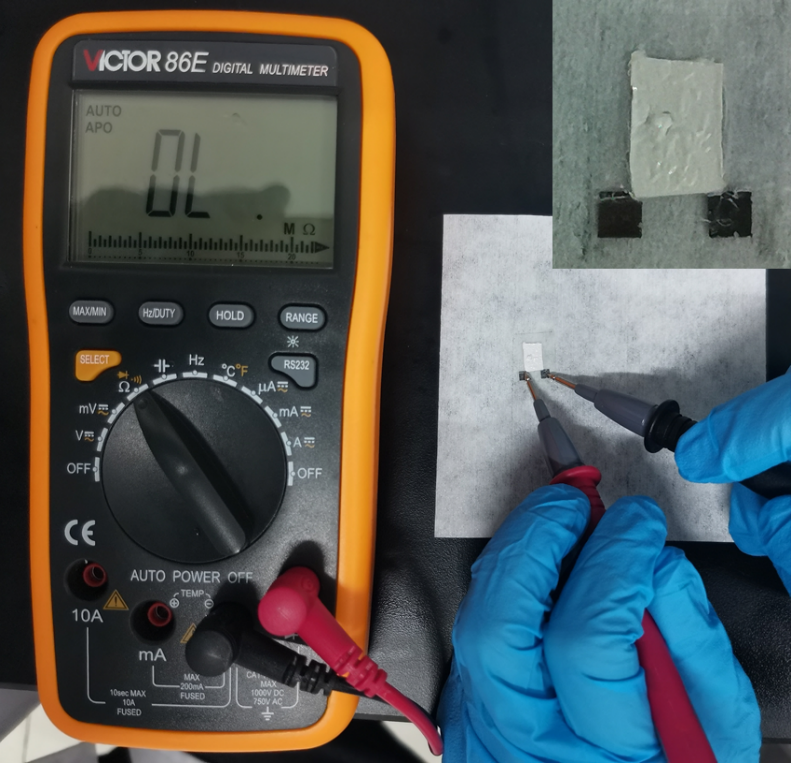


**Figure S16** Photograph of the BiOCl-based flexible gas sensor.

**Figure S17** Linear fitting of the sensing response as a function of gas concentration in the range of 0.1 ppm to 0.8 ppm for the BiOCl/Bi_2_Se_3_ sensor under bent conditions. The detection limit was extrapolated from the linear calibration curve when the response was equal to 3 times of the average noise (0.03%), which was extracted from the baseline.

Table S1 Comparison of the operating temperature, sensing response, bending condition of flexible NO_2_ gas sensors.

| **Flexible sensors** | **Temperature** | NO_2_ conc. (ppm) | Response under bended condition | Bending condition | **Ref.** |
| --- | --- | --- | --- | --- | --- |
| Bi_2_Se_3_/BiOCl | RT | 0.1 | 12.5% | 7.5 mm bending radius | This work |
|  |  | 1 | 74.6% |  |  |
|  |  | 5 | 101.4% |  |  |
| rGO nanofibrous mesh fabric | RT | 1 | 0.009% | 1 mm bending radius | [[7](#_ENREF_7)] |
| rGO | RT | 5 | 11.5% | 30° bending angle | [[8](#_ENREF_8)] |
| 3D SnS_2_/ rGO | RT | 8 | 50% ^a^ | 120° bending angle | [[9](#_ENREF_9)] |
| Ti_3_C_2_T_x_/ZnO | RT | 100 | 30% ^a^ | 120° bending angle | [[10](#_ENREF_10)] |
| WO_3_ NPs-MWCNTs-RGO | RT | 5 | 16.7% ^a^ | 90° bending angle | [[11](#_ENREF_11)] |
| MoS_2_ | RT | 25 | 90% ^a^ | 7 mm bending radius | [[12](#_ENREF_12)] |
| PbS colloidal quantum dots | RT | 50 | 21.5% ^a^ | 70° bending angle | [[13](#_ENREF_13)] |
| SnO_2_/rGO | RT | 4 | 15% ^a^ | 150° bending angle | [[14](#_ENREF_14)] |

RT means room temperature.

^a^ denotes the estimated value from the figures in references

**Figure S18** Responses of Bi_2_Se_3_/BiOCl-based sensors under different bending radius to various NO_2_ concentrations.

**Figure S19** Responses of a typical Bi_2_Se_3_/BiOCl-based sensor before and after 100 times of repeated bending at various NO_2_ concentrations.

**Reference**

[1] M. Segall, P. J. Lindan, M. a. Probert*, et al.*, "First-principles simulation: Ideas, illustrations and the CASTEP code," *Journal of Physics: Condensed Matter*, vol. 14, no. 11, pp. 2717-2744, 2002.

[2] J. P. Perdew, K. Burke, M. Ernzerhof, "Generalized gradient approximation made simple," *Physical Review Letters*, vol. 77, no. 18, article 3865, 1996.

[3] J. White, D. Bird, "Implementation of gradient-corrected exchange-correlation potentials in Car-Parrinello total-energy calculations," *Physical Review B*, vol. 50, no. 7, article 4954, 1994.

[4] W. Zhou, H. Zhu, J. A. Yarmoff, "Termination of single-crystal Bi_2_Se_3_ surfaces prepared by various methods," *Physical Review B*, vol. 94, no. 19, article 195408, 2016.

[5] J. Jeon, K. Yu, J. Kim*, et al.*, "Observation of optical absorption correlated with surface state of topological insulator," *Physical Review B*, vol. 100, no. 19, article 195110, 2019.

[6] B. Li, L. Shao, R. Wang*, et al.*, "Interfacial synergism of Pd-decorated BiOCl ultrathin nanosheets for the selective oxidation of aromatic alcohols," *Journal of Materials Chemistry A*, vol. 6, no. 15, pp. 6344-6355, 2018.

[7] H. J. Park, W.-J. Kim, H.-K. Lee*, et al.*, "Highly flexible, mechanically stable, and sensitive NO_2_ gas sensors based on reduced graphene oxide nanofibrous mesh fabric for flexible electronics," *Sensors and Actuators B: Chemical*, vol. 257, pp. 846-852, 2018.

[8] P.-G. Su, H.-C. Shieh, "Flexible NO_2_ sensors fabricated by layer-by-layer covalent anchoring and in situ reduction of graphene oxide," *Sensors and Actuators B: Chemical*, vol. 190, pp. 865-872, 2014.

[9] J. Wu, Z. Wu, H. Ding*, et al.*, "Flexible, 3D SnS_2_/reduced graphene oxide heterostructured NO_2_ sensor," *Sensors and Actuators B: Chemical*, vol. 305, article 127445, 2020.

[10] Z. Yang, L. Jiang, J. Wang*, et al.*, "Flexible resistive NO_2_ gas sensor of three-dimensional crumpled MXene Ti_3_C_2_T_x_/ZnO spheres for room temperature application," *Sensors and Actuators B: Chemical*, vol. 326, article 128828, 2021.

[11] U. Yaqoob, A. S. M. I. Uddin, G.-S. Chung, "A high-performance flexible NO_2_ sensor based on WO_3_ NPs decorated on MWCNTs and RGO hybrids on PI/PET substrates," *Sensors and Actuators B: Chemical*, vol. 224, pp. 738-746, 2016.

[12] Y. Zhao, J. G. Song, G. H. Ryu*, et al.*, "Low-temperature synthesis of 2D MoS_2_ on a plastic substrate for a flexible gas sensor," *Nanoscale*, vol. 10, no. 19, pp. 9338-9345, 2018.

[13] H. Liu, M. Li, O. Voznyy*, et al.*, "Physically flexible, rapid-response gas sensor based on colloidal quantum dot solids," *Advanced Materials*, vol. 26, no. 17, pp. 2718-2724, 2014.

[14] J. Wu, Z. Wu, H. Ding*, et al.*, "Three-dimensional graphene hydrogel decorated with SnO_2_ for high-performance NO_2_ sensing with enhanced immunity to humidity," *ACS Applied Materials & Interfaces*, vol. 12, no. 2, pp. 2634-2643, 2020.
